# Adolescent mental health interventions: a narrative review of the positive effects of physical activity and implementation strategies

**DOI:** 10.3389/fpsyg.2024.1433698

**Published:** 2024-06-27

**Authors:** Zhaojin Li, Jie Li, Jianda Kong, Zhilin Li, Rui Wang, Fugao Jiang

**Affiliations:** ^1^Department of Physical Education, Qufu Normal University, Jining, China; ^2^Langfang Traditional Chinese Medicine Hospital, Langfang, China

**Keywords:** adolescent mental health, physical activity, suicide prevention, psychological intervention, social skills, emotional regulation

## Abstract

**Introduction:**

The psychological well-being of adolescents is a global concern due to increasing societal pressures and mental health issues. Physical activity is known to enhance physical health and has potential benefits for mental health, including reducing symptoms of anxiety and depression, boosting self-esteem, and improving social skills. This narrative review explores how physical activity can serve as an intervention to help adolescents manage psychological stress and prevent mental health issues.

**Methods:**

An extensive literature search was conducted using databases such as PubMed, PsycINFO, Web of Science, and Scopus. Keywords included “adolescent mental health,” “physical activity,” “psychological intervention,” “types of exercise,” “anxiety,” “depression,” “self-esteem,” “social skills,” and “emotional regulation.” Studies were included based on relevance, peer-reviewed status, and involvement of adolescent populations. Data were extracted and analyzed qualitatively, focusing on the psychological impacts of different types of physical activity. Sixty one articles were eventually included.

**Results and conclusion:**

The review identified multiple studies highlighting the positive effects of various physical activities on adolescent mental health. Aerobic exercises were found to improve mood and cognitive function, strength training reduced depressive symptoms and increased self-efficacy, team sports enhanced social skills and a sense of community, and mind–body practices like yoga and tai chi improved stress management and emotional regulation. The findings suggest that physical activity can play a significant role in promoting adolescent mental health. Implementation strategies in school and community settings, including integrating physical activity into school curricula, offering diverse activity options, training professional instructors, encouraging family and community involvement, and regular monitoring and evaluation, are recommended. Future research should address limitations such as sample diversity and long-term effects. This narrative review underscores the importance of physical activity in enhancing adolescent mental health. Effective implementation strategies and multi-sector collaboration are essential for maximizing the benefits of physical activity interventions.

## 1 Introduction

Due to the increasing social pressures and mental health issues, adolescent mental health has become a growing global concern. According to the World Health Organization (WHO), depression is one of the leading causes of illness and disability among adolescents, and suicide is the second leading cause of death for individuals aged 15–29 (World Health Organization, 2021; Klaufus et al., 2022; Korczak et al., 2023). Recent studies indicate that approximately one in seven adolescents aged 10–19 globally suffers from mental health issues, with depression and anxiety being the most prevalent problems (Klaufus et al., 2022). These mental health issues are highly prevalent among adolescents, with depression and anxiety accounting for about 40% of adolescent mental health problems worldwide (UNICEF, 2021; World Health Organization, 2021). Adolescence is a critical period marked by significant physical, emotional, and social changes, making individuals particularly vulnerable to mental health issues (World Health Organization, 2021). Therefore, timely identification and treatment of these issues are crucial for the long-term health and well-being of adolescents (O'Leary, 2021).

The increase in adolescent mental health problems can be attributed to various factors, including academic pressure, the influence of social media, family dynamics, and socioeconomic conditions (Buli et al., 2021; Källmén and Hallgren, 2021; Gautam et al., 2024). The COVID-19 pandemic has further exacerbated these issues, leading to increased isolation, anxiety, and uncertainty among young people. Research indicates that the pandemic has significantly worsened the mental health status of adolescents. Adolescents have commonly experienced anxiety and loneliness due to social isolation, disrupted routines, and uncertainty about the future, contributing to the rise in mental health issues (Huang and Ougrin, 2021; Meneguzzo et al., 2024). Consequently, addressing adolescent mental health issues is not only a medical challenge but also a societal responsibility, necessitating comprehensive and multi-faceted intervention strategies.

Physical activity is widely recognized for its substantial benefits in promoting physical health. Over the past decades, increasing research has focused on the positive impact of physical activity on mental health. Regular physical activity has been shown to improve mental health by reducing symptoms of anxiety and depression, enhancing self-esteem, and strengthening social skills (Singh et al., 2023). Physical activity also serves as an effective stress-relief mechanism, enhancing individual coping strategies, alleviating psychological stress, and helping prevent suicidal behaviors (Nightingale et al., 2024). Additionally, different types of physical activities offer various psychological benefits. Aerobic exercise has been found to enhance mood and cognitive function by increasing cerebral blood flow and promoting the release of endorphins (Yao et al., 2021). Strength training is associated with the reduction of depressive symptoms and the improvement of self-efficacy (Gordon et al., 2018). Team sports not only improve physical health but also cultivate social skills and community awareness, which are crucial for adolescent development (Easterlin et al., 2019). Mind–body exercises such as yoga and tai chi promote emotional regulation and stress management by balancing the sympathetic and parasympathetic nervous systems (Zou et al., 2018). Despite the recognized benefits, systematically implementing physical activity as a psychological intervention for adolescents requires further exploration. Many schools and communities lack structured programs to incorporate physical activity into adolescents’ daily lives, necessitating evidence-based strategies to promote and sustain participation in physical activities.

This narrative review aims to synthesize existing literature on the role of physical activity in enhancing adolescent mental health. By analyzing the impact of different types of physical activities on mental health, this paper proposes implementation strategies and discusses their effectiveness in various settings and conditions. Additionally, this review identifies gaps in current research and suggests directions for future studies to better understand and utilize physical activity as a tool for promoting adolescent mental health.

## 2 Methods

### 2.1 Search strategy and database selection

To ensure the comprehensiveness and timeliness of our review, we conducted extensive literature searches across multiple databases, including but not limited to the following: (i) PubMed: This database encompasses a vast array of research literature in the fields of medicine and life sciences. (ii) PsycINFO: Specializing in literature on psychology and behavioral sciences. (iii) Web of Science: Contains high-impact research literature across multiple disciplines. (iv) Scopus: Widely covers literature in science, technology, medicine, and social sciences.

### 2.2 Keywords

During the literature search process, we employed a range of keywords related to the research topic to ensure comprehensive coverage of all relevant studies. These keywords included: “adolescent mental health,” “physical activity,” “psychological intervention,” “types of exercise,” “anxiety,” “depression,” “self-esteem,” “social skills,” and “emotional regulation.”

### 2.3 Inclusion and exclusion criteria

To ensure that the included literature was of high quality and relevance, we established the following inclusion and exclusion criteria:

Inclusion Criteria: (i) Studies published in peer-reviewed journals. (ii) Studies involving adolescent populations (aged 10–19) or other populations that can be referenced in this review. (iii) Studies investigating the impact of physical activity on mental health. (iv) Studies providing clear research methods and results.

Exclusion Criteria: (i) Studies not involving physical activity or adolescent mental health. (ii) Conference abstracts, review articles, and unpublished studies. (iii) Studies not providing sufficient data or detailed methods. (iv) Non-English literature.

### 2.4 Data extraction and analysis

Upon determining the studies to be included, we conducted systematic data extraction and analysis. The data extracted included but were not limited to the following: (i) Study Design: Including cross-sectional studies, longitudinal studies, randomized controlled trials, etc. (ii) Sample Characteristics: Including sample size, age, gender, geographical location, etc. (iii) Intervention Type: Detailed descriptions of different types of physical activities, such as aerobic exercise, strength training, team sports, and mind–body practices. (iv) Main Findings: Summarizing the key conclusions of each study, particularly the specific impacts of physical activity on mental health. (v) Method Quality: Evaluating the quality of research methods, including randomization, blinding, control group setup, etc.

We employed both qualitative and quantitative analyses to compare and synthesize the results of different studies, identifying commonalities and differences. Additionally, we addressed potential biases and limitations within the studies and provided detailed discussion in the results section.

### 2.5 Search results

**Figure 1:**
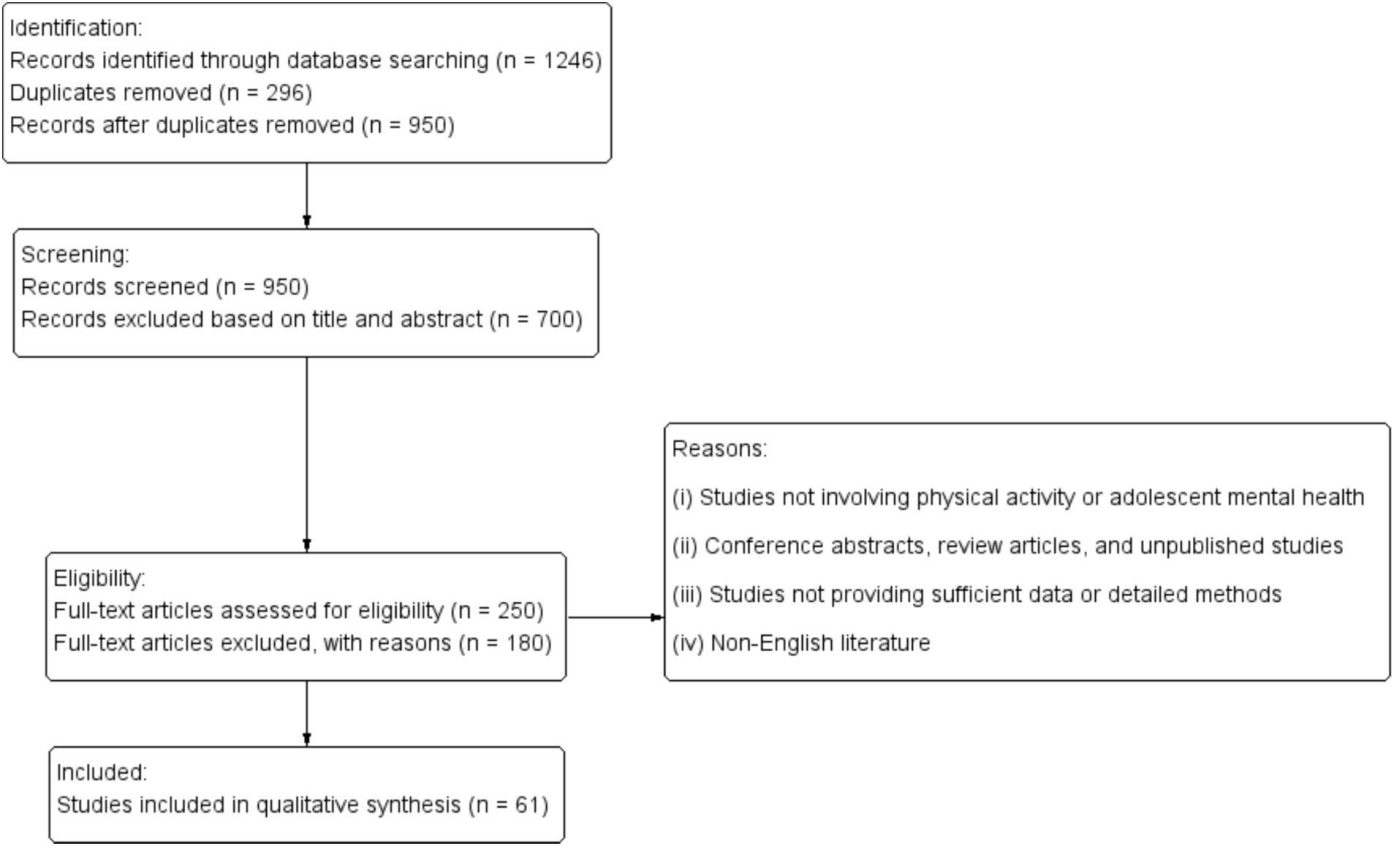
The specific search results and screening process are shown in the PRISMA flowchart.

The initial search produced a total of 1,246 articles. After removing duplicates, 950 articles remained. These articles were screened based on title and abstract, and 700 articles that did not meet the inclusion criteria were excluded. The remaining 250 articles were reviewed in full text, of which 180 were excluded because they did not meet the inclusion criteria or lacked sufficient data. Finally, 61 articles were included in this narrative review. The specific search results and screening process are shown in the PRISMA flowchart (Figure 1).

### 2.6 Data extraction and analysis

Data extraction was carried out systematically, focusing on study design, sample characteristics, types of physical activity, key findings and methodological quality. Qualitative and quantitative analyses were used to compare and synthesize findings across studies, identifying commonalities and differences. Potential biases and limitations of the studies are discussed in the findings section.

## 3 Results

### 3.1 Physical activity and adolescent mental health

#### 3.1.1 Psychological health benefits

**Figure 2:**
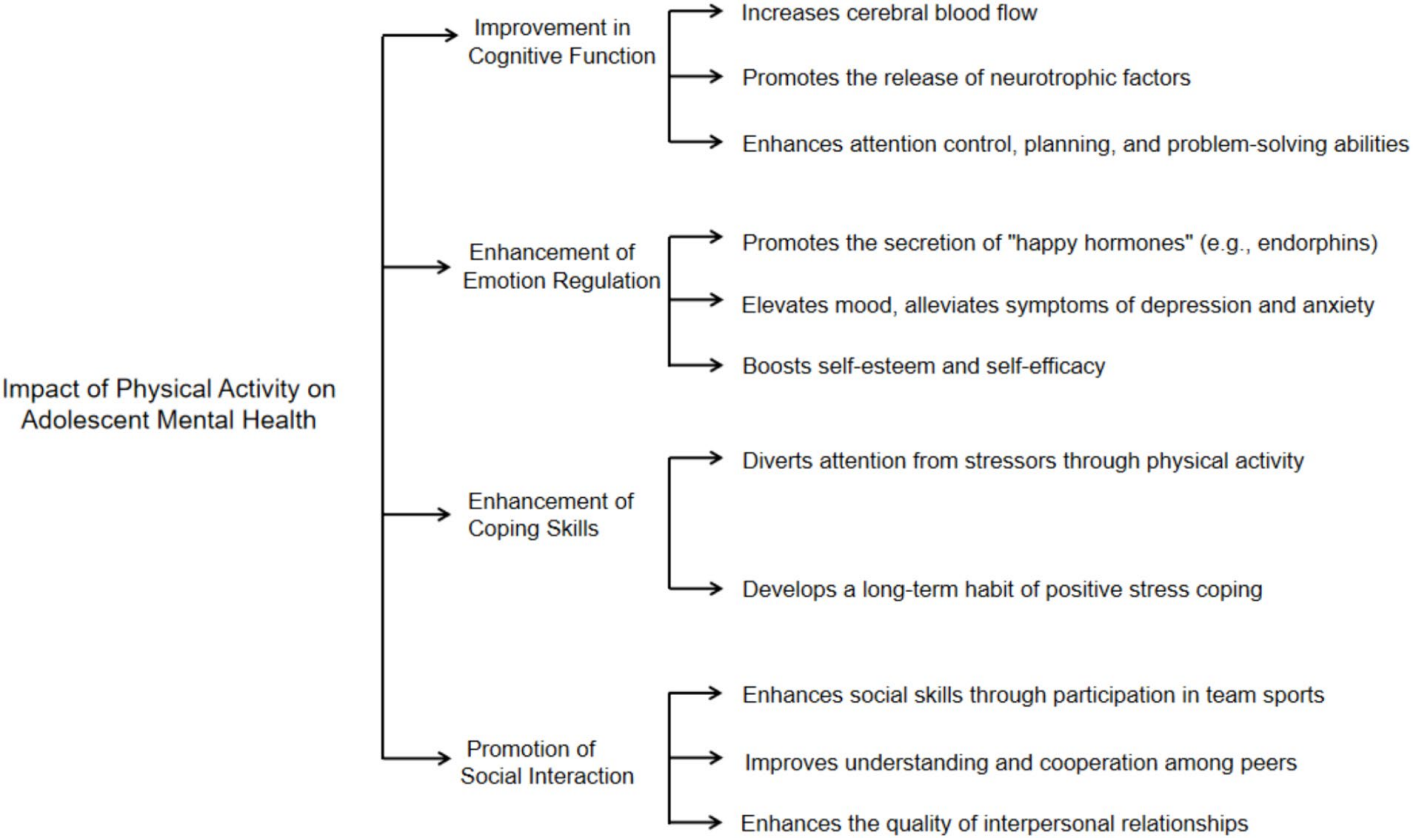
The specific advantages of sports activities on the mental health of adolescents (Goldberg et al., 2002; Anderson and Shivakumar, 2013; Prickett et al., 2015; Nakano et al., 2016; Moeijes et al., 2018; Guddal et al., 2019; Logan et al., 2019; Hu et al., 2020, 2023; Teuber et al., 2024).

The benefits of Physical Activity on the psychological health of adolescents are multifaceted, involving cognition, emotion, and social behavior. Regular participation in Physical Activity can significantly improve the emotional state of adolescents, reduce symptoms of anxiety and depression, and enhance psychological well-being. Specifically, Physical Activity enhances cognitive function by increasing cerebral blood flow and promoting neurotrophic factor release, which is vital during adolescence, a critical period for brain development (Goldberg et al., 2002; Prickett et al., 2015; Nakano et al., 2016). Additionally, it enhances emotion regulation by boosting the secretion of endorphins, alleviating symptoms of depression and anxiety, and improving self-esteem and self-efficacy (Anderson and Shivakumar, 2013; Hu et al., 2020, 2023). Physical Activity also serves as an effective tool for stress relief, helping adolescents divert focus from stressors and cultivate long-term positive coping habits (Hu et al., 2023; Teuber et al., 2024). Furthermore, participation in team sports fosters social skills, cooperation, and understanding, thereby enhancing psychological well-being, self-esteem, and social identity among adolescents (Moeijes et al., 2018; Guddal et al., 2019; Logan et al., 2019). Figure 2 illustrates the specific advantages of sports activities on the mental health of adolescents.

##### 3.1.1.1 Improvement in cognitive function

Physical Activity improve cognitive function by enhancing cerebral blood flow and promoting the release of neurotrophic factors. This is particularly important for adolescents as it is a critical period for their brain development and cognitive function. For example, studies have shown that regular aerobic exercise can enhance adolescents’ executive functions, including better attention control, planning, and problem-solving abilities (Goldberg et al., 2002; Nakano et al., 2016). Furthermore, research indicates that the effects of Physical Activity on the brain include increased cerebral blood flow and oxygen metabolism, thereby promoting the release of neurotrophic factors. These factors collectively contribute to enhancing cognitive function. This is particularly significant in adolescents, as this stage is crucial for brain development, and by enhancing neuronal connectivity and function, Physical Activity can effectively promote the development of cognitive abilities (Goldberg et al., 2002; Prickett et al., 2015).

##### 3.1.1.2 Enhancement of emotion regulation

Physical activity contributes to the enhancement of mood by promoting the secretion of “happy hormones” such as endorphins, aiding in mood elevation and alleviation of symptoms related to depression and anxiety. Studies indicate that exercise can increase the activity of endogenous opioid substances (such as β-endorphins) in the brain, which play a crucial role in regulating emotions and emotional responses. This mechanism is known as the endorphin hypothesis, which suggests that the mood enhancement and anxiety reduction after exercise are due to the release of β-endorphins and their binding to brain receptors (Anderson and Shivakumar, 2013; Hu et al., 2020). Furthermore, exercise can also enhance self-esteem and self-efficacy among adolescents, both of which are crucial factors in positive emotion regulation. Research suggests that physical activity not only aids in neurogenesis but also improves brain function and mental health by increasing the levels of brain-derived neurotrophic factor (BDNF) (Hu et al., 2020, 2023). These mechanisms collectively make physical activity an effective means of improving mental health.

##### 3.1.1.3 Enhancement of coping skills

Physical activity is regarded as an effective tool for stress release. Studies suggest that through exercise, adolescents can divert their focus from stressors (such as academic pressure and interpersonal relationships) to physical Activity, aiding in alleviating feelings of tension and anxiety (Hu et al., 2023; Teuber et al., 2024). Moreover, regular engagement in physical activity also helps in cultivating a habit of positive stress coping over the long term, thereby reducing stress-related psychological issues (Hu et al., 2023). Regular exercise not only improves the psychological well-being of adolescents but also promotes their cognitive and emotional development (Hu et al., 2023; Teuber et al., 2024).

##### 3.1.1.4 Promotion of social interaction

Participation in physical Activity, especially team sports, can enhance the social skills of adolescents, improve understanding and cooperation through interaction with peers, and enhance the quality of interpersonal relationships. This social interaction is crucial for the social identity and psychological health development of adolescents.

According to research, participating in team sports contributes to the development of social skills among adolescents, enhancing their self-esteem and social abilities. Such involvement not only fosters cooperation and understanding among adolescents but also elevates their levels of psychological well-being. For instance, studies indicate that adolescents engaged in team sports experience fewer mental health issues such as anxiety and depression compared to their non-participating peers (Moeijes et al., 2018; Logan et al., 2019). Additionally, team sports, by fostering friendships and strengthening social support networks, contribute to enhancing the sense of social identity and self-worth among adolescents (Guddal et al., 2019). These social interactions play a crucial role in the comprehensive development of adolescents. In conclusion, team sports not only contribute to the physical health of adolescents but also significantly promote their social skills and psychological well-being.

#### 3.1.2 Types of exercise and psychological effects

**Figure 3:**
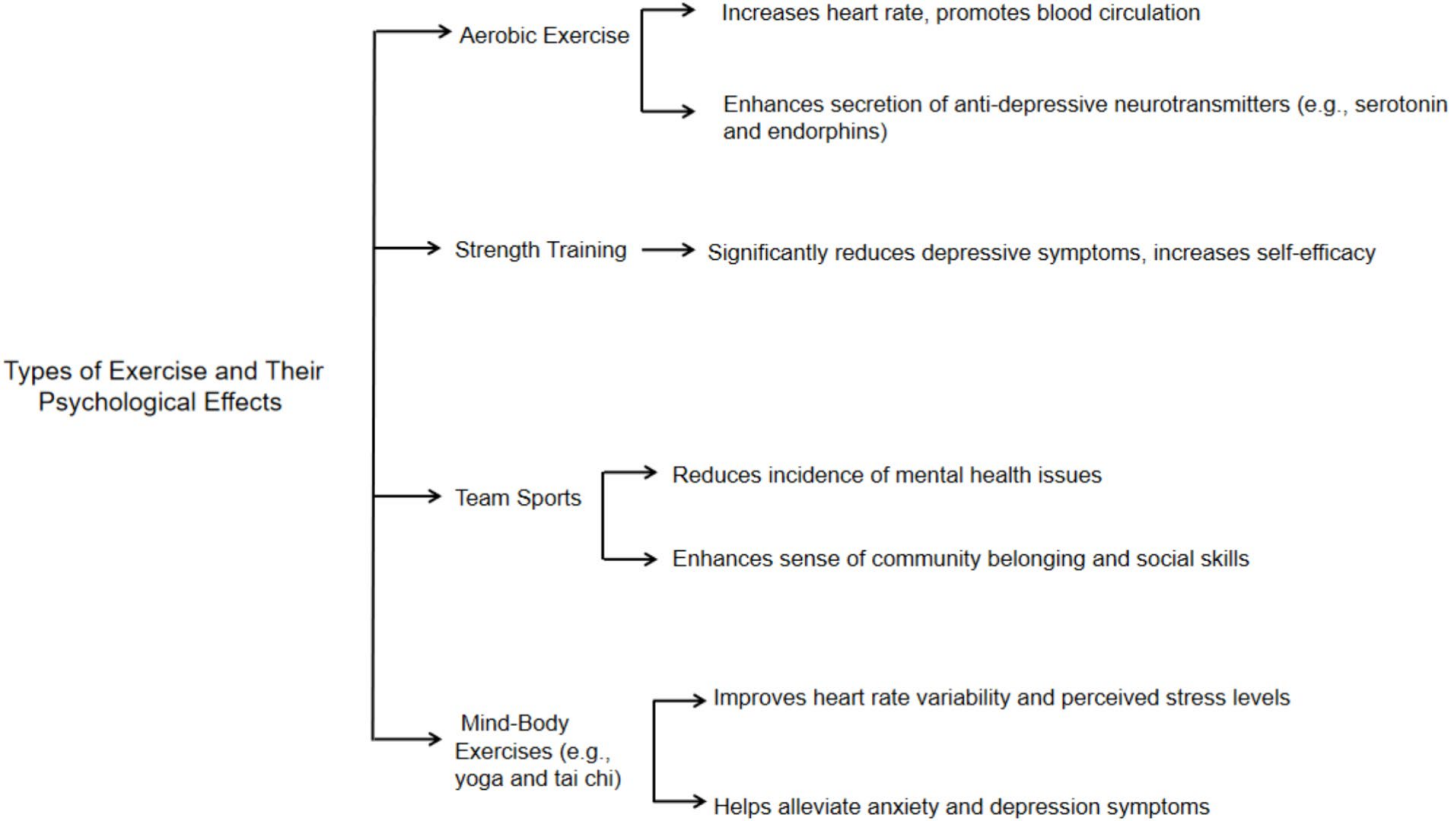
The potential effects of different types of exercise on adolescent mental health (Foley and Fleshner, 2008; Eime et al., 2013; Gordon et al., 2018; Zou et al., 2018; Easterlin et al., 2019; Liu et al., 2021; Xie et al., 2021; Yao et al., 2021; Hoffmann et al., 2022).

Different types of exercise positively impact mental health, each offering unique benefits. Aerobic exercises like running, swimming, and cycling enhance cardiovascular function, cognitive abilities, and emotional well-being by increasing heart rate and promoting the release of mood-enhancing neurotransmitters like serotonin and endorphins, effectively reducing anxiety and depression symptoms (Foley and Fleshner, 2008; Xie et al., 2021; Yao et al., 2021). Strength training has also been shown to reduce depressive symptoms and improve self-efficacy, making individuals feel more capable of handling daily challenges (Gordon et al., 2018). Participation in team sports significantly lowers the incidence of mental health problems, improves social skills, and enhances a sense of community belonging (Eime et al., 2013; Easterlin et al., 2019; Hoffmann et al., 2022). Mind–body exercises such as yoga and tai chi are effective in stress and emotion management, improving heart rate variability, reducing perceived stress, and alleviating anxiety and depressive symptoms (Zou et al., 2018; Liu et al., 2021). In summary, various forms of exercise play a crucial role in promoting psychological health, particularly for adolescents. This section explores the distinct impacts of these exercise types on adolescent mental health, with a detailed overview provided in Figure 3.

##### 3.1.2.1 Aerobic exercise

Aerobic exercises such as running, swimming, and cycling have a positive impact on mental health. Research indicates that aerobic exercise significantly increases heart rate, promotes blood circulation, and enhances the secretion of anti-depressive neurotransmitters in the brain, such as serotonin and endorphins, effectively reducing symptoms of anxiety and depression and improving overall emotional state (Xie et al., 2021). Studies have also found that moderate aerobic exercise not only improves cardiovascular function but also enhances cognitive function and emotional status, which is particularly beneficial for the psychological health of the elderly (Yao et al., 2021). Furthermore, the benefits of exercise on the brain include improving its ability to cope with stress, increasing dopamine and endorphin levels, which can improve mood and induce feelings of happiness (Foley and Fleshner, 2008).

In summary, aerobic exercise is an effective way to improve mental health, particularly as an adjunctive treatment for conditions such as depression.

##### 3.1.2.2 Strength training

The positive impact of strength training on mental health has been supported by some research. For example, a meta-analysis found that strength training significantly reduces depressive symptoms, indicating its potential as an alternative or adjunctive treatment for depression (Gordon et al., 2018). Additionally, regular physical activity, including strength training, is associated with increased self-efficacy, which refers to an individual’s belief in their ability to control the environment and achieve goals (Gordon et al., 2018). By improving physical strength and endurance, adolescents also feel psychologically stronger to face the challenges of daily life.

##### 3.1.2.3 Team sports

Team sports have significant benefits for the psychological health of adolescents. Studies have shown that adolescents participating in team sports have a lower incidence of mental health problems compared to those who do not participate. For example, a study involving over 11,000 children and adolescents in the United States found that organized team sports participation significantly reduces the occurrence of mental health difficulties (Hoffmann et al., 2022). Additionally, another study also showed that participants in team sports have better mental health outcomes in adulthood, such as lower rates of depression and anxiety (Easterlin et al., 2019).

Participation in team sports not only helps improve social skills but also enhances a sense of community belonging, which is crucial for improving adolescents’ social abilities and satisfaction with interpersonal relationships. Through teamwork, adolescents can learn how to communicate effectively and coordinate interpersonal relationships, skills that are essential for their overall development (Eime et al., 2013; Hoffmann et al., 2022). In conclusion, team sports are an effective way to promote the psychological and social health of adolescents by providing social support and enhancing a sense of community.

##### 3.1.2.4 Mind–body exercises

The comprehensive effects of mind–body exercises such as yoga and tai chi have been confirmed in multiple studies, particularly in the adolescent population regarding stress and emotion management. For example, a systematic review analyzed multiple randomized controlled trials, showing that these mind–body practices significantly improve heart rate variability parameters and perceived stress levels, suggesting that they may alleviate stress by regulating the balance between the sympathetic and parasympathetic nervous systems (Zou et al., 2018). Another review examined the psychological effects of tai chi and qigong use in adolescents, finding that these practices help alleviate anxiety and depressive symptoms and may reduce cortisol levels, indicating that tai chi and qigong can be effective methods for improving the psychological well-being of adolescents (Yao et al., 2021). These studies emphasize the importance of yoga and tai chi practices in promoting both physical and mental health, especially during adolescence, a critical stage of development.

#### 3.1.3 Negative impact of physical activity on mental health

While physical activity has numerous benefits for mental health, it is also important to acknowledge potential negative impacts if not properly managed. One such issue is exercise addiction, characterized by an unhealthy obsession with physical activity that can lead to both physical and psychological harm. Hall et al. (2007) identified motivational antecedents of obligatory exercise, such as achievement goals and multidimensional perfectionism, which can contribute to exercise addiction. Their study highlights that individuals driven by perfectionistic tendencies and high achievement goals are at a higher risk of developing an addiction to exercise (Hall et al., 2007). Similarly, Allegre et al. (2006) discussed various definitions and measures of exercise dependence, emphasizing the complexity of diagnosing and addressing this issue. They noted that exercise dependence can lead to negative consequences such as physical injuries, social isolation, and psychological distress (Allegre et al., 2006).

Another potential negative impact is the development of eating disorders and body image disorders. For instance, Kanayama et al. (2001) noted that the use of ‘body image’ drugs and excessive physical activity can lead to serious psychosomatic problems. Their study indicated that individuals engaging in high levels of exercise to enhance their body image might develop body dysmorphic disorders, leading to severe mental health issues (Kanayama et al., 2001). Additionally, Davis et al. (1995) found a correlation between obsessive-compulsive behaviors and high levels of physical activity in individuals with anorexia nervosa. This suggests that excessive exercise can exacerbate symptoms of anorexia and other eating disorders (Davis et al., 1995). Furthermore, the abrupt cessation of physical activity can lead to withdrawal symptoms such as depressive mood and fatigue. Berlin et al. (2006) studied the effects of exercise withdrawal and found that individuals who suddenly stop exercising may experience significant decreases in mood and increased fatigue, likely due to decreased physical fitness levels and the sudden absence of previously beneficial physiological effects (Berlin et al., 2006).

These findings emphasize the need for a balanced and sustainable approach to physical activity, ensuring that the positive benefits are maximized while minimizing the potential for negative outcomes. It is crucial for practitioners and educators to monitor for signs of unhealthy behavior patterns and to provide appropriate guidance and support.

### 3.2 Intervention strategies and effects

In implementing interventions to utilize Physical Activity for adolescent psychological well-being, key strategies include integrating it into school curriculum, offering diverse activity options, training qualified instructors, fostering family and community involvement, and regularly evaluating intervention effects. Empirical research underscores the positive impact of Physical Activity on adolescents, particularly through team sports and varied activities, with rigorous methodologies needed to address challenges like sample representativeness and activity control. By addressing these challenges and employing effective strategies, policymakers and educators can promote adolescent psychological health through Physical Activity. Table 1 provides recommendations for appropriate implementation strategies for physical activity to promote mental health in children and adolescents.

**Table 1:** Suggestions for appropriate implementation strategies of physical activity for the mental health of children and adolescents.

Intervention strategy	Implementation details	Psychological effects	Social effects	Physical effects	References
Integrating physical activity into school curriculum	- Establish regular physical education classes - Enrich after-school Physical Activity	- Lower levels of anxiety and depression - Higher self-esteem	- Enhanced teamwork skills and social interaction - Sense of belonging	- Improved physical health	Eime et al. (2016), Guddal et al. (2019), Hu et al. (2021) and Stabelini Neto et al. (2022)
Offering a diverse range of physical activity	- Provide team sports, individual competitive sports, and low-intensity activities Accommodate diverse needs and interests	- Enhanced self-management and self-efficacy - Stress reduction and relaxation through low-intensity activities like yoga and tai chi	- Broadened social networks and acceptance of diversity - Improved social skills through team sports	- Enhanced flexibility and balance	Allender et al. (2006), Logan et al. (2019), Eather et al. (2023) and Grauduszus et al. (2023)
Training professional sports teachers and coaches	- Qualify teachers and coaches in sports techniques and mental health education - Incorporate mental health education into sports activities	- Improved understanding of teamwork and fair competition - Reduced stigma around mental health issues	- Enhanced leadership qualities and conflict resolution skills - Increased support-seeking behaviors	–	Bissett et al. (2020) and Hebard et al. (2021)
Encouraging family and community participation	- Organize community sports events and family sports days - Promote participation of family and community members	- Strengthened family bonds - Increased sense of belonging	- Fostering community cohesion	- Enhanced cardiovascular health through family activities	Laird et al. (2016), Super et al. (2018), Van der Veken et al. (2020) and Davis et al. (2021)
Monitoring and evaluating intervention effects	- Utilize surveys, psychological assessments, and physical health examinations - Regularly assess participation, psychological health status, and changes among adolescents - Adjust strategies based on feedback	- Greater awareness of psychological well-being - Enhanced self-awareness	- Improved academic performance through regular physical activity	- Reduced risk of chronic diseases through physical fitness assessments	Camacho-Miñano et al. (2011), Eime et al. (2016) and Stabelini Neto et al. (2022)

#### 3.2.1 Intervention strategies and implementation

To effectively utilize physical Activity for intervening in adolescent psychological stress and preventing suicidal behavior, the key lies in devising scientific strategies and ensuring the practical implementation of these strategies. The following are several strategies for implementing sports interventions.

##### 3.2.1.1 Integrating physical activity into school curriculum

Schools are the primary venues for adolescents’ activities for the majority of their time, and integrating Physical Activity into daily curriculum proves to be an effective approach to enhance adolescent engagement. By establishing regular physical education classes and enriching after-school Physical Activity, schools can encourage student participation while educating them about the psychological benefits of Physical Activity. Research indicates that adolescents who regularly participate in Physical Activity demonstrate better psychological health, including lower levels of anxiety and depression, as well as higher self-esteem (Guddal et al., 2019; Hu et al., 2021). Furthermore, the school environment provides an ideal setting to promote these activities since adolescents spend a significant amount of time in school, and the structured environment of schools facilitates the incorporation of Physical Activity into their daily lives (Eime et al., 2016; Stabelini Neto et al., 2022).

##### 3.2.1.2 Offering a diverse range of physical activity

Adolescents vary greatly in interests and capabilities, and providing a variety of sports options can meet a broader range of needs. This includes team sports, individual competitive sports, and low-intensity activities such as yoga and tai chi, allowing all students to choose the activities that best suit their personal preferences and physical conditions. Such diverse sports options help promote the physical, psychological, and social health development of adolescents (Allender et al., 2006; Logan et al., 2019; Grauduszus et al., 2023).

Studies indicate that participating in both team sports and individual competitive sports can enhance the physical fitness and psychological health levels of adolescents (Grauduszus et al., 2023). For example, team sports not only enhance physical health but also improve teamwork and social skills (Eather et al., 2023). On the other hand, individual competitive sports contribute to improving self-management and self-efficacy (Eather et al., 2023). Low-intensity activities such as yoga and tai chi also offer significant benefits. They are not only suitable for students of different physical abilities but also effectively reduce stress and anxiety, promoting holistic development of body and mind (Allender et al., 2006) to accommodate the diverse needs and interests of different students.

##### 3.2.1.3 Training professional sports teachers and coaches

Well-qualified teachers and coaches are not only capable of instructing correct sports techniques but can also incorporate elements of mental health education into their activities. Coaches can enhance education in teamwork and fair competition during training sessions, aiding youth in establishing positive social interactions and self-awareness.

The role of sports coaches in mental health education is crucial. Through deliberate communication, destigmatizing mental health issues, and establishing supportive relationships, coaches can significantly impact athletes’ mental health and help-seeking behaviors (Hebard et al., 2021). Some programs have begun to explore adapting mental health interventions to the sports setting, allowing coaches to implement these interventions during training, thereby extending the influence of mental health professionals. The implementation of these interventions can not only assist athletes in addressing mental health issues but also promote their overall psychological well-being and social interactions (Bissett et al., 2020).

##### 3.2.1.4 Encouraging family and community participation

Support from families and communities is crucial for the sustained involvement of adolescents in Physical Activity. Organizing community sports events and family sports days not only enhances the motivation of young people to participate but also fosters social support networks at the family and community levels, providing more emotional and material support to the youth. These activities offer benefits for physical health and also promote social inclusion and mental well-being by enhancing a sense of belonging, self-esteem, and self-efficacy (Super et al., 2018; Van der Veken et al., 2020; Davis et al., 2021). Community sports programs and family sports days create a safe, supportive, and cooperative environment by encouraging the participation of family and community members, aiding in the holistic development of the youth (Laird et al., 2016).

##### 3.2.1.5 Monitoring and evaluating intervention effects

To ensure the effectiveness of sports activity interventions, it is necessary to regularly monitor and evaluate the participation, psychological health status, and changes among adolescents. Utilizing surveys, psychological assessment tools, and physical health examinations can aid educators and policymakers in understanding the impact of interventions and adjusting strategies based on feedback.

Regular monitoring and evaluation of adolescents’ participation in Physical Activity and changes in their psychological health are crucial. Research indicates that the school environment provides an ideal platform to promote adolescents’ physical activity and psychological health through sports courses and extracurricular activities (Camacho-Miñano et al., 2011; Stabelini Neto et al., 2022). Systematic assessment tools, such as surveys, psychological health assessment instruments, and physical health examinations, can provide a comprehensive understanding of the effects of interventions and serve as a basis for adjusting strategies (Eime et al., 2013; Jacob et al., 2021). Furthermore, diversified intervention measures, including support from parents and peers as well as digital health programs, have been proven to have positive effects on enhancing the levels of physical activity and psychological health among adolescents (Demetriou et al., 2019; Stabelini Neto et al., 2022). Through these means, educators and policymakers can better understand the needs and changes of adolescents, thereby formulating and optimizing sports activity intervention strategies to enhance their promotive effect on adolescent health development.

#### 3.2.2 Effect evaluation and empirical research

##### 3.2.2.1 Overview of empirical research

In recent years, numerous studies have focused on the positive impacts of Physical Activity on adolescent psychological health. For instance, a cross-national study investigated the effects of various types of Physical Activity on depressive symptoms, finding that adolescents who regularly engage in sports display lower symptoms of depression and anxiety (Chillón et al., 2010). Moreover, compared to individual sports, team sports appear to be more effective in enhancing social skills and self-esteem, possibly due to increased peer interaction and cooperation. Research indicates significant improvements in social skills and self-esteem among adolescents participating in team sports, closely linked to the social support and sense of belonging provided by these Activity (Eime et al., 2013). Additionally, team sports can reduce social anxiety and feelings of isolation, promoting a better social self-concept (Eime et al., 2013). These positive psychological and social effects are primarily due to the cooperation and interaction among team members, which help individuals find their value and confidence within the team environment (Bojanić et al., 2019; Marschin and Herbert, 2021).

##### 3.2.2.2 Methods and tools

To systematically assess the impact of Physical Activity on adolescent psychological health, researchers have employed various methods including surveys, psychological assessment tools, and physiological markers. These tools help researchers collect data and evaluate changes in psychological stress, anxiety, depression, and self-esteem.

These studies demonstrate the positive effects of Physical Activity on adolescent psychological health. For instance, during the COVID-19 pandemic, physical exercise helped alleviate anxiety and depression, enhancing well-being (Ai et al., 2021). Additionally, systematic reviews and meta-analyses indicate that Physical Activity can effectively improve the psychological health of adolescents, including reducing psychological stress and enhancing self-esteem (Khor et al., 2021; Forthal et al., 2022). Researchers also use physiological markers (such as heart rate and blood pressure) to supplement psychological assessments, providing a comprehensive understanding of the specific impacts of Physical Activity on adolescent psychological health (Valentine et al., 2024). By integrating multiple methods, researchers can more accurately assess the multifaceted impact of Physical Activity on adolescent psychological health.

##### 3.2.2.3 Evaluation of intervention effects

Studies indicate that Physical Activity can significantly enhance adolescent psychological health. By comparing data before and after interventions, many studies consistently find that Physical Activity improve adolescents’ psychological states, reducing negative emotions such as depression and anxiety, and enhancing overall psychological well-being. Furthermore, the frequency and intensity of participation in Physical Activity are positively correlated with the degree of improvement in psychological health (Pascoe et al., 2020; Marconcin et al., 2022; Peng et al., 2022).

A systematic review revealed that the more frequently adolescents engage in Physical Activity, the more significant the improvements in their psychological health. Particularly, moderate to high-intensity Physical Activity have a substantial effect in reducing depressive symptoms and enhancing life satisfaction (Marconcin et al., 2022). Another meta-analysis further supports this view, noting a significant positive correlation between the frequency and intensity of physical activity and the extent of improvement in psychological health (Peng et al., 2022). In summary, regular participation in Physical Activity can significantly enhance the psychological health of adolescents, with higher frequencies and intensities of Activity leading to better outcomes.

##### 3.2.2.4 Challenges and limitations

Despite many positive findings, these studies also face challenges and limitations that need to be addressed in future research to enhance the generalizability and interpretability of the results. Specific challenges include: (i) Sample representativeness: Most existing studies rely on samples from specific regions or groups, which may limit the universality of the findings. Future research should expand the sample base to include diverse geographical locations, cultural backgrounds, and socioeconomic statuses to enhance the representativeness and applicability of the results. (ii) Participant self-selection bias: Many studies rely on volunteer participants, which may lead to self-selection bias as individuals inclined to participate in Physical Activity might already possess higher motivation and better psychological health. This bias can affect the objectivity and universality of the results. (iii) Control of activity type and frequency: It is often challenging to strictly control the type and frequency of Physical Activity in studies, which can impact the accuracy of the results. Different types and intensities of Physical Activity may have significant variations in their impact on psychological health, thus precise control and recording of these variables are crucial for analyzing specific effects. (iv) Lack of randomized controlled trials: There is a scarcity of randomized controlled trials in current research, which are the gold standard in scientific studies for establishing causal relationships. Future research should employ this study design, randomly assigning participants to experimental and control groups to reduce bias and enhance the reliability of the findings.

To address these issues, future research should adopt more rigorous methodological designs, such as using broader and more diverse samples, conducting randomized controlled trials, and more precisely recording and controlling the types of Activity and their frequencies. Through these improvements, researchers can more accurately assess the impact of Physical Activity on adolescent psychological health and provide a more robust scientific basis for related policies and practices.

## 4 Conclusion and outlook

### 4.1 Limitations of current research and future directions

Despite the current research highlighting the positive impacts of Physical Activity on adolescent psychological health, there are several limitations that guide future research directions: (i) Sample diversity and representativeness: Current studies often rely on data from specific regions or populations. Future research needs to expand the sample scope to include adolescents from various cultural, economic, and geographic backgrounds to enhance the universality and transferability of the results; (ii) Long-term follow-up studies: Most studies focus on short-term psychological health effects and lack long-term follow-up data. Future studies should consider the sustained effects of long-term interventions and how these effects evolve over time; (iii) Innovation in intervention methods: Current research is largely centered on traditional Physical Activity, such as running and swimming. Future studies might explore the impact of more innovative Physical Activity (e.g., virtual reality sports, interactive fitness games) on adolescent psychological health; (iv) Elucidation of psychological effect mechanisms: The current understanding of how Physical Activity influence adolescent psychological states through physiological and psychological mechanisms is still incomplete. Future research should delve deeper into these mechanisms, for example, through interdisciplinary studies involving neurobiology, psychology, and sociology.

### 4.2 Recommendations for practice and policy

Based on the findings of the research, the following recommendations are made for practice and policy implementation: (i) Integration of Physical Activity into school curricula: Educational sectors should consider Physical Activity as an integral part of the daily school curriculum, not only as a tool for enhancing physical health but also as a strategy for preventing and improving mental health issues; (ii) Multi-sectoral collaboration: It is recommended that health, education, and social services sectors collaborate to design and implement sports-based mental health promotion programs, especially targeting disadvantaged youth groups; (iii) Policy support and resource allocation: Governments should provide necessary resources, such as funding, facilities, and professional training, to ensure the effective implementation of sports activity programs; (iv) Public education and advocacy: Strengthen public education and advocacy about the mental health benefits of Physical Activity to increase participation awareness and motivation among parents and adolescents; and (v) Data monitoring and evaluation system: Establish a comprehensive data monitoring and evaluation system to regularly assess the effects of sports activity interventions, allowing for timely adjustments and optimization of strategies.

## Author contributions

ZhaL: Conceptualization, Data curation, Formal analysis, Funding acquisition, Investigation, Methodology, Project administration, Resources, Software, Supervision, Validation, Visualization, Writing – original draft, Writing – review & editing. JL: Conceptualization, Data curation, Formal analysis, Funding acquisition, Investigation, Methodology, Project administration, Resources, Software, Supervision, Validation, Visualization, Writing – original draft, Writing – review & editing. JK: Conceptualization, Data curation, Formal analysis, Funding acquisition, Investigation, Methodology, Project administration, Resources, Software, Supervision, Validation, Visualization, Writing – original draft, Writing – review & editing. ZhiL: Conceptualization, Data curation, Formal analysis, Funding acquisition, Investigation, Methodology, Project administration, Resources, Software, Supervision, Validation, Visualization, Writing – original draft, Writing – review & editing. RW: Conceptualization, Data curation, Formal analysis, Funding acquisition, Investigation, Methodology, Project administration, Resources, Software, Supervision, Validation, Visualization, Writing – original draft, Writing – review & editing. FJ: Conceptualization, Data curation, Formal analysis, Funding acquisition, Investigation, Methodology, Project administration, Resources, Software, Supervision, Validation, Visualization, Writing – original draft, Writing – review & editing.
